# Effect of medium pH on chemical selectivity of oxalic acid biosynthesis by *Aspergillus niger* W78C in submerged batch cultures with sucrose as a carbon source

**DOI:** 10.1007/s11696-017-0354-x

**Published:** 2017-12-05

**Authors:** Ewa Walaszczyk, Waldemar Podgórski, Małgorzata Janczar-Smuga, Ewelina Dymarska

**Affiliations:** 1 Department of Biotechnology and Food Analysis, Institute of Chemistry and Food Technology, Faculty of Engineering and Economics, Wroclaw University of Economics, Komandorska 118-120, 53-345 Wrocław, Poland; 2 Department of Animal Food Technology, Institute of Chemistry and Food Technology, Faculty of Engineering and Economics, Wroclaw University of Economics, Komandorska 118-120, 53-345 Wrocław, Poland; 3 Department of Human Nutrition, Faculty of Health Sciences and Physical Education, The Witelon State University of Applied Sciences in Legnica, Sejmowa 5A, 59-220 Legnica, Poland

**Keywords:** Oxalic acid, *Aspergillus niger*, Biosynthesis, pH, Chemical selectivity

## Abstract

The pH of the medium is the key environmental parameter of chemical selectivity of oxalic acid biosynthesis by *Aspergillus niger*. The activity of the enzyme oxaloacetate hydrolase, which is responsible for decomposition of oxaloacetate to oxalate and acetate inside the cell of the fungus, is highest at pH 6. In the present study, the influence of pH in the range of 3–7 on oxalic acid secretion by *A. niger* W78C from sucrose was investigated. The highest oxalic acid concentration, 64.3 g dm^−3^, was reached in the medium with pH 6. The chemical selectivity of the process was 58.6% because of the presence of citric and gluconic acids in the cultivation broth in the amount of 15.3 and 30.2 g dm^−3^, respectively. Both an increase and a decrease of medium pH caused a decrease of oxalic acid concentration. The obtained results confirm that pH 6 of the carbohydrate medium is appropriate for oxalic acid synthesis by *A. niger*, but the chemical selectivity of the process described in this paper was high in comparison to values reported previously in the literature.

## Introduction

Oxalic acid (ethanedioic acid, OA), the first in the homologous series of aliphatic dicarboxylic acids, is used in various industrial areas, such as metal surface treatment and textile manufacture and processing (Sawada and Murakami 2000). Although the greatest demand for it is in the pharmaceutical industry, the acid has been reported to be widely used in the chemical industry (Abraham et al. 2014; Kundu et al. 2015; Lesmana and Wu 2014), in biohydrometallurgy (Qu et al. 2015; Vakilchap et al. 2016) and in environmental protection (Bahaloo-Horeh and Mousavi 2017; Bahaloo Horeh et al. 2016; Mi et al. 2015). Its wide applications emerge from its chemical properties to complex or bond metals to form metal complexes or salts in mostly insoluble compounds.

The metal oxalates—potassium, calcium, sodium, manganese or iron salts—are widely distributed in the plant kingdom. They play significant roles in heavy metal detoxification, calcium level regulation and defence against fungal or insect infections (Gadd et al. 2014; Sawada and Murakami 2000). Because of its wide presence in vegetable food products, oxalic acid may be used instead of strong inorganic acids such as sulphuric or nitric acid as anti-browning and preservation agents in fruit and vegetable storage (Cefola and Pace 2015; Li et al. 2016; Ruíz-Jiménez et al. 2014; Wang et al. 2016). There is social resistance to the use of chemically synthesised additives in the food industry, thus other natural ways of their production are being sought. Oxalic acid is biologically produced widely by various microorganisms, among which the filamentous fungus *Aspergillus niger* is recognised as the best producer (Musiał et al. 2006; Podgórski and Leśniak 2003; Strasser et al. 1994). The biosynthesis of oxalic acid in *A. niger* is possible exclusively by splitting of oxaloacetate formed directly from pyruvate to oxalate and acetate by the enzyme oxaloacetate hydrolase (OAH) [E.C. 3.7.1.1.] (Gadd et al. 2014; Kubicek et al. 1988; Pedersen et al. 2000a). The secretion of oxalic acid is a reaction to environmental changes: it is produced when the ambient pH is near neutral, and its role is to lower the pH quickly to prevent competing microorganisms from growing (Andersen et al. 2009; Poulsen et al. 2012). That is why the optimal activity of OAH is in the pH range of 5–8 (Pedersen et al. 2000b; Ruijter et al. 1999).

Sucrose was found to be the most suitable carbon source for oxalic acid biosynthesis by *A. niger*, but the large amount of gluconic and citric acids produced then by the fungus causing low chemical selectivity of the processes was indicated as the main disadvantage of using this substrate (Cameselle et al. 1998; Musiał et al. 2005; Strasser et al. 1994; Walaszczyk et al. 2014, 2017). In the experiments described in this paper, both the oxalic acid concentration and the chemical selectivity of its biosynthesis are high in comparison to those reported earlier in the literature despite using sucrose as a sole carbon source. The paper also presents a detailed description of changes in concentration of secreted acids during cultivation of *A. niger* W78C at different pH values.

The aim of the study was to determine the influence of medium pH on chemical selectivity of the biosynthesis of oxalic acid from sucrose by *A. niger* W78C in submerged batch cultures.

## Experimental

### Materials and medium preparation

The experiments were carried out with *A. niger* W78C strain from the collection of the Department of Biotechnology and Food Analysis, Wroclaw University of Economics (Poland), selected in previous research (Walaszczyk et al. 2015) as the strain with the highest oxalic acid production selectivity from sucrose.

The only carbon and energy source for the microorganism was sucrose in the form of white sugar. The synthetic medium, used in previous experiments (Walaszczyk et al. 2014, 2017), consisted (per 1 dm^−3^) of: sucrose 125 g, NH_4_NO_3_ 1.89 g, KH_2_PO_4_ 0.32 g, MgSO_4_·7H_2_O 0.64 g, ZnSO_4_·7H_2_O 0.97 mg, CuSO_4_·5H_2_O 0.86 mg, FeSO_4_·7H_2_O 1.64 mg, MnSO_4_·H_2_O 1.02 mg, and distilled water. After sterilization, the medium was inoculated with spores of *A. niger* in the amount of about 3 × 10^5^ per 1 cm^3^ of the medium.

### Culture conditions

The experiments were carried out for 7–12 days at 30 °C in a stirred tank reactor, Biostat A (Sartorius AG, Germany), with the working volume of 5 dm^3^ filled with 4 dm^3^ of the medium. The aeration and agitation were fixed at 6 dm^3^ dm^−3^ h^−1^ and 600 min^−1^, respectively. The pH was adjusted to a required level (3, 4, 5, 6 or 7) before sterilisation and regulated automatically by the addition of 10 M KOH from the beginning of secretion of acids. The exact moment in each experiment was determined based on the courses of the curves of oxygen uptake and carbon dioxide output. The secretion of the acids started when the volumetric oxygen uptake rate exceeded the volumetric carbon dioxide evolution rate. It took place between 44 and 50 h of the process. The end of the process was set by the lack of increase of total acidity of the medium measured titrimetrically with 0.1 M NaOH in the presence of phenolphthalein.

### Analytical methods

The samples of the culture broth were taken for analysis once daily in the amount of 25 cm^3^. The concentrations of organic acids were measured by HPLC using a Rezex ROA Organic Acid column (Phenomenex, USA) coupled to a UV detector at 210 nm and an RI detector (Perkin Elmer, USA). The column was eluted with 0.0025 M H_2_SO_4_ at room temperature and a flow rate of 0.5 ml min^−1^.

The statistical analysis of results was done with MS Excel 2013 and Statistica 12.0 (StatSoft, Inc., USA). Results were considered statistically significant at a level of *p* ≤ 0.05.

## Results and discussion

*Aspergillus niger* cultivated in medium containing carbohydrates produces mostly three organic acids—citric, oxalic and gluconic—and the medium pH is one of the most significant factors influencing this process (Papagianni 2007). The strong oxalic acid is synthesised when the environmental pH is slightly acidic or neutral to lower the pH in the shortest possible time to prevent the growth of competing microorganisms, mostly bacteria. The weaker citric acid with its buffering properties is secreted when the ambient pH is low, to maintain it and prevent it from rising. Gluconic acid production is optimal at pH 5.5, and it is not aimed at acidifying the medium but at storing all the glucose as a compound unavailable to competing microorganisms. The created gluconate is later reused by *A. niger* as a carbon source to produce other acids (Andersen et al. 2009; Poulsen et al. 2012).

**Fig. 1:**
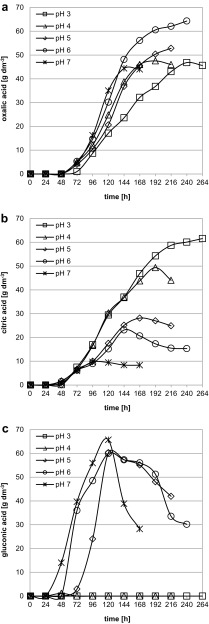
The courses of oxalic (**a**), citric (**b**) and gluconic (**c**) acids concentration during cultivation of *A. niger* W78C in defined medium with sucrose at different pH values

In the present study, the medium pH of 3, 4, 5, 6 and 7 was examined. The courses of oxalic (a), citric (b) and gluconic (c) acid concentrations obtained in medium with different pH values are presented in Fig. 1. Oxalic acid was detected in all medium variants from the third day (72 h) of the process and its amount grew rapidly during the next few days. The highest concentration, 64.3 g dm^−3^, was reached at 240 h at pH 6. Both the increase and the decrease of medium pH caused the decrease of oxalic acid concentration (Fig. 1a). These findings are in accordance with the results described by other authors who investigated the influence of pH on oxalic acid production in media with glucose or lactose as the carbon source (Bohlmann et al. 1998; Kubicek et al. 1988; Ruijter et al. 1999). They indicated that the most suitable medium pH for this process is 6, but the amounts of product they obtained were low, between 8.0 and 21.3 g dm^−3^. When sucrose was a carbon source and the medium pH was 6, the concentration of oxalic acid reported in the literature was higher, between 33.8 and 64.2 g dm^−3^ (Cameselle et al. 1998; Foryś and Podgórski 2004; Podgórski and Leśniak 2003; Strasser et al. 1994; Walaszczyk et al. 2015) or even 70.8 g dm^−3^ (Walaszczyk et al. 2017), depending on medium composition and culture conditions.

In all the experiments, apart from oxalic acid, *A. niger* secreted accompanying acids: either only citric acid (pH 3) or citric and gluconic acids (pH 4, 5, 6, 7) (Fig. 1b, c). Citric acid was detected in small amounts (0.2–1.7 g dm^−3^) on the second day of cultivation (48 h). As expected (Papagianni 2007), the lower the pH of the medium was, the greater was the amount of citric acid produced and the later was the maximum concentration reached; the highest amount of this metabolite, 61.6 g dm^−3^, was detected at pH 3 at the 264th hour of the process (Fig. 1b). The opposite relation was found in the case of gluconic acid. This acid was present in high amounts in media with higher pH, i.e., 5, 6 and 7 (Fig. 1c). With increasing medium pH, its production began earlier and grew faster, and a higher concentration was reached; the highest amount of gluconic acid, 65.7 g dm^−3^, was found at pH 7 at the 120th hour of cultivation. At the same time of the process, the highest concentrations of this by-product, 60.3 and 60.0 g dm^−3^, were detected in media with pH levels of 5 and 6, respectively. In the medium variant with the lowest tested pH, i.e., 3, there was no gluconic acid present, and at pH 4, a small amount of the acid was found solely at the 48th hour. Probably either the enzyme glucose oxidase [E.C. 1.1.3.4.] involved in glucose transformation to gluconic acid was not active at lower pH values [its maximum activity is reported to be at pH 5.5 (Andersen et al. 2009; Papagianni 2007)] or the entire amount of carbon source was used to produce citric and oxalic acids (Fig. 1a, b).

In most of the experiment variants (all beside the medium with pH 3) the concentration of accompanying citric and gluconic acids decreased after reaching the maximum (Fig. 1b, c). The acids were probably reused by *A. niger* as carbon sources to produce oxalic acid because the pH was constantly automatically regulated and this acid’s role is to lower the pH quickly. The same cultivation courses and conclusions were reported by other authors (Cameselle et al. 1998; Musiał et al. 2005; Poulsen et al. 2012; Strasser et al. 1994; Walaszczyk et al. 2014, 2017). It is worth mentioning that the final concentration of gluconic acid in all experiments presented in this paper was lower than that of oxalic acid, in contrast to some of the other results of oxalic acid biosynthesis from sucrose (Cameselle et al. 1998; Musiał et al. 2005; Strasser et al. 1994).

**Fig. 2:**
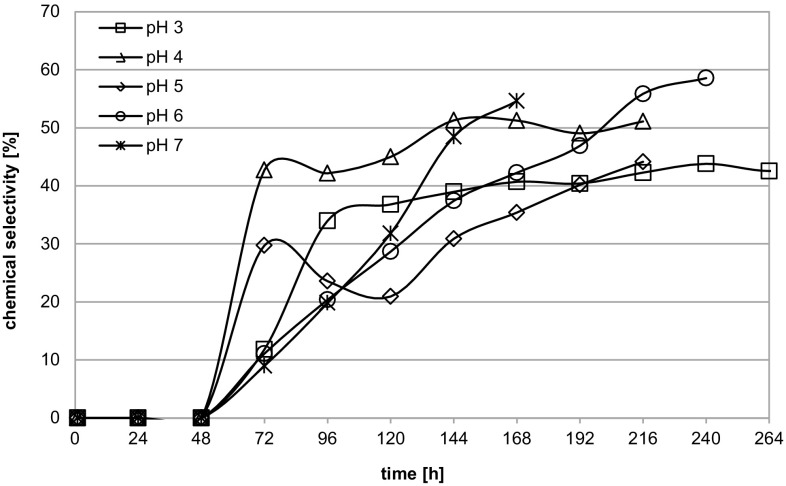
The course of chemical selectivity of oxalic acid biosynthesis by *A. niger* W78C cultivated in defined medium with sucrose at different pH values

Because *A. niger* cultivated in sucrose defined media beside oxalic acid also secretes citric and gluconic acids, as described earlier, the selectivity of oxalic acid biosynthesis is one of the most important parameters for evaluation of the results. The courses of chemical selectivity expressed as the relation of the amount of oxalic acid to the amount of all the acids secreted by *A. niger*, at different medium pH values, are shown in Fig. 2. The chemical selectivity of most processes grew constantly from the beginning of oxalic acid presence in the cultivation broth. The exception was the cultivation at pH 5 when at 96 h and at 120 h the gluconic acid concentration grew significantly in relation to its value at 72 h (Fig. 1c), so the chemical selectivity dropped. The maximum value of chemical selectivity, 58.6%, was reached at 240 h at pH 6 (Fig. 2). Such chemical selectivity of oxalic acid biosynthesis by *A. niger* from sucrose is higher than that described by other authors: Strasser et al. (1994) reported selectivity of 38.7%, Cameselle et al. (1998) 40.8% and Musiał et al. (2005) only 27.1%. All these authors detected amounts of gluconic acid higher than oxalic acid in their cultivation liquids and indicated that as the main disadvantage of using sucrose as a carbon source for this process. Only in our own previous research (Walaszczyk et al. 2015, 2017) was the chemical selectivity of conducted processes of oxalic acid bioproduction from sucrose higher, 69.8–72.1%.

## Conclusions

The presented results show that medium pH is an important factor influencing secretion of organic acids by *A. niger* W78C in submerged batch cultures with sucrose as the carbon source. The study findings confirm that the most suitable medium pH for oxalic acid biosynthesis from sucrose is 6. Despite the large amounts of accompanying acids, mostly gluconic, both the final concentration of the product and the chemical selectivity of the process were high. Further research will be conducted to eliminate co-production of unwanted by-products, so the chemical selectivity of oxalic acid biosynthesis could significantly rise.
